# Comparative Genome Analysis Reveals *Cis*-Regulatory Elements on Gene-Sized Chromosomes of Ciliated Protists

**DOI:** 10.3389/fmicb.2022.775646

**Published:** 2022-02-21

**Authors:** Weibo Zheng, Huan Dou, Chao Li, Saleh A. Al-Farraj, Adam Byerly, Naomi A. Stover, Weibo Song, Xiao Chen, Lifang Li

**Affiliations:** ^1^Laboratory of Marine Protozoan Biodiversity and Evolution, Marine College, Shandong University, Weihai, China; ^2^School of Life Sciences, Ludong University, Yantai, China; ^3^Institute of Evolution and Marine Biodiversity, Ocean University of China, Qingdao, China; ^4^Zoology Department, College of Science, King Saud University, Riyadh, Saudi Arabia; ^5^Department of Computer Science, Bradley University, Peoria, IL, United States; ^6^Department of Biology, Bradley University, Peoria, IL, United States

**Keywords:** ciliates, *Pseudokeronopsis carnea*, *Pseudokeronopsis flava*, phylogenomics, nanochromosome

## Abstract

Gene-sized chromosomes are a distinct feature of the macronuclear genome in ciliated protists known as spirotrichs. These nanochromosomes are often only several kilobase pairs long and contain a coding region for a single gene. However, the ways in which transcription is regulated on nanochromosomes is still largely unknown. Here, we generated macronuclear genome assemblies for two species of *Pseudokeronopsis* ciliates to better understand transcription regulation on gene-sized chromosomes. We searched within the short subtelomeric regions for potential *cis*-regulatory elements and identified distinct AT-rich sequences conserved in both species, at both the 5’ and 3’ end of each gene. We further acquired transcriptomic data for these species, which showed the 5’ *cis*-regulatory element is associated with active gene expression. Gene family evolution analysis suggests nanochromosomes in spirotrichs may originated approximately 900 million years ago. Together our comparative genomic analyses reveal novel insights into the biological roles of *cis*-regulatory elements on gene-sized chromosomes.

## Introduction

Gene-sized chromosomes (nanochromosomes) are an intriguing genetic architecture found in a subset of ciliates, one of the most diverse clades of unicellular eukaryotes. Ciliates contain two distinct types of nuclei, called the micro- and macronucleus, which are both present in the cell throughout its vegetative life cycle (Yan et al., 2017; Jiang et al., 2019; Sheng et al., 2021). During sexual reproduction (conjugation), zygotic micronuclear (MIC) chromosomes are fragmented into somatic macronuclear (MAC) chromosomes through a series of genome wide rearrangements (Chalker and Yao, 2011; Chen et al., 2014; Zhao et al., 2019, 2020; Sheng et al., 2020). For spirotrichous ciliates including *Oxytricha*, *Stylonychia*, *Uroleptopsis*, *Euplotes* and *Strombidium*, this fragmentation is extensive, resulting in a MAC genome composed of more than 10,000 telomere-capped chromosomes (Steinbrück et al., 1981; Prescott, 2000; Swart et al., 2013; Zheng et al., 2018; Chen et al., 2019; Li et al., 2021). These linear nanochromosomes are usually smaller than mitochondrial chromosomes and can carry one or few genes and can be few hundred base pairs up to several kilobases in size (Swart et al., 2013; Zhang et al., 2021). These general features make spirotrichous ciliates excellent models to study chromosomal architecture and functions (Zhao et al., 2021; Zheng et al., 2021).

The *cis*-regulatory elements (CREs, e.g., promoter, enhancer, and insulator) needed for transcription regulation in eukaryotes are often located in intergenic regions (Levine and Tjian, 2003). The existence and nature of *cis*-regulatory elements, and the methods of gene regulation in ciliates in general, are poorly understood. Furthermore, since nanochromosomes contain only very short regions outside of the coding sequence, the space to harbor CREs is limited. Surprisingly, studies of other spirotrich genomes have not immediately revealed the presence of any conserved, recognizable sequences or patterns that could be tied to the regulation of gene expression (Swart et al., 2013; Chen et al., 2019; Li et al., 2021; Zheng et al., 2021).

**FIGURE 1:**
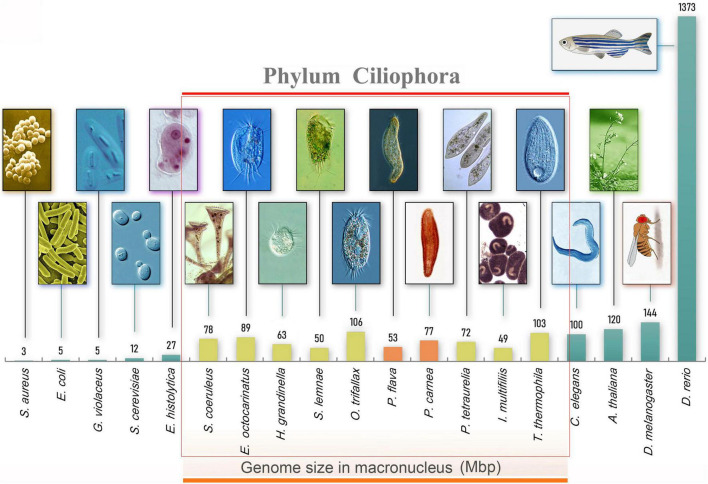
Morphology and genome size of model organisms including ciliates. From left to right, bacteria (*Staphylococcus aureus* and *Escherichia coli*), cyanobacteria (*Gloeobacter violaceus*), yeast (*Saccharomyces cerevisiae*), amoeboid protists (*Entamoeba moshkovskii*), ciliated protists (*Ichthyophthirius multifiliis*, *Stylonychia lemnae*, *Oxytricha trifallax*, *Pseudokeronopsis flava*, *Halteria grandinella*, *Paramecium tetraurelia*, *Pseudokeronopsis carnea*, *Stentor coeruleus*, *Euplotes octocarinatus*, *Tetrahymena thermophila*), nematode (*Caenorhabditis elegans*), thale cress (*Arabidopsis thaliana*), fruit fly (*Drosophila melanogaster*), and zebrafish (*Danio rerio*).

To help elucidate the mechanism of transcription regulation on gene-sized chromosomes, we carried out deep genomic sequencing and assembly for the MAC genomes of two new spirotrichous ciliates, *Pseudokeronopsis carnea* and *Pseudokeronopsis flava*. *Pseudokeronopsis* species have long been recognized and studied for their distinct cell shape and fascinating pigment colors (Song et al., 2004; Baek et al., 2011) (Figure 1 and Supplementary Table 1). Like other spirotrichs, *Pseudokeronopsis* cells are large and can provide abundant DNA, making them ideal prospects for in-depth genetic studies (Dong et al., 2020; Luo et al., 2021). To date these types of studies have been limited by a lack of genomic data, and many features of *Pseudokeronopsis* genomes, including the presence of nanochromosomes, have been unknown until now. Combining both genomic and transcriptomic data, we searched the subtelomeric regions of their compact chromosomes for potential conserved CREs. Using a variety of genome evolution analyses, we reveal the origin of gene-sized chromosomes in spirotrichs and the regulatory elements they harbor.

## Materials and Methods

### Cell Culture and Sample Preparation

*Pseudokeronopsis carnea* and *P. flava* cells were isolated from a freshwater pond in Baihuayuan Park (36°04′N, 120°22′E), Qingdao, China. Species were initially determined by morphological features and later confirmed by sequencing their SSU-rRNA genes. A single cell was picked, washed, and cultured in flasks using filtered and autoclaved pond water. Cells were incubated with rice grains at 23°C for 21 days, then collected using a glass micropipette under a stereomicroscope. Genomic DNA was extracted using the MagAttract HMW DNA kit (QIAGEN, #67563, Germany). A DNA library was constructed with NEBNext DNA Library Prep Master Mix Set for Illumina (NEB, United States) following the manufacturer’s instructions. RNA extraction was performed with the RNeasy Plus Mini Kit (Qiagen, Germany) following the manufacturer’s instructions. The RNA libraries were generated using NEBNext Ultra RNA Library Prep Kit for Illumina (NEB, United States) following the manufacturer’s instructions.

### Illumina Sequencing and Genome Assembly

Pair-end 150 bp sequencing reads were performed on the Illumina Hiseq 2500 platform, producing 20 Gb and 10 Gb of clean data for the DNA and RNA libraries, respectively. Genomes were assembled using SPAdes v3.12 (Bankevich et al., 2012) (parameters: -k 21,33,55,77 –careful). Contigs with low coverage (< 2×) or small size (< 200 bp) were removed. QUAST v5.0.2 (Gurevich et al., 2013) was used to measure genomic statistics including GC content and N50. RSEM v1.3.3 (Li and Dewey, 2011) was used to calculate the sequencing depth of each contig. For homologous gene annotation, genomic contigs were aligned with protein sequences from the SWISS-PROT database using BLASTX v2.3.0 (Camacho et al., 2009) (parameters: evalue = 1e-5, querygenecode = 6). Gene model annotation information was extracted using a custom Perl script and used to train AUGUSTUS v2.5.5 (Stanke et al., 2006) (parameters: –species = pseudokeronopsis –min_intron_len 15,39) for gene model prediction. RNA-seq reads were assembled into transcripts using rnaSPAdes v3.11.1 (Bushmanova et al., 2019) and aligned with the genome assembly by BLAT v3.6 (Kent, 2002) to optimize the gene models. Predicted genes without start and stop codons were filtered out using a custom Perl script. The RNA-seq reads were mapped to genome contigs using Tophat2 v2.0.10 (Kim et al., 2013). The mapped read count of each gene was measured by featureCounts v1.6.1 (Liao et al., 2013). Potential *cis*-regulatory sequence motifs were searched within subtelomeric regions using MEME v5.3.3 (Bailey et al., 2015). The sequence motifs identified were visualized using WebLogo 3 (Crooks et al., 2004). Frequency of stop codon usage (TAA, TGA, and TAG) was measured from the homolog sequence alignment between the CDS or transcript sequences of each species and the ciliate protein library using BLASTX v2.3.0 (parameter: evalue = 1e-5), as previously described (Pan et al., 2019).

### Phylogenomic Analysis and Genome Evolution

A total of 238 orthogroups were identified among 31 ciliates (see Supplementary Table 2) using OrthoFinder and were aligned using mafft (Emms and Kelly, 2019). The concatenated ortholog sequence alignment dataset was used for phylogenomic analysis on CIPRES Science Gateway server v3.3 (Miller et al., 2010). RAxML-HPC2 v8.2.9 (Stamatakis, 2014) under LG model of amino acid substitution (Γ distribution + F, four rate categories, 1,000 bootstrap replicates) was used to perform maximum likelihood (ML) analysis. PhyloBayes MPI 1.5a (Lartillot et al., 2009) (CAT-GTR model + Γ distribution, four independent chains, 4,000 generations with 10% burn-in, convergence Maxdiff < 0.3) was used to perform Bayesian inference (BI) analysis. The phylogenetic tree was visualized using MEGA v7.0.20 (Kumar et al., 2016). The time of speciation was estimated using r8s (Sanderson, 2003) and corrected using calibration times obtained from the TimeTree database (Hedges et al., 2006). Computational analysis of gene family evolution (CAFE) (De Bie et al., 2006) was performed to identify gene families that have undergone significant expansion or contraction. Gene families were annotated against the Gene Ontology (GO) database using InterProscan (Jones et al., 2014). R package clusterprofiler (Yu et al., 2012) was used to conduct an enrichment analysis of expanded and contracted gene families.

## Results and Discussion

### Compact Genome Architecture and Reassigned Stop Codons

**FIGURE 2:**
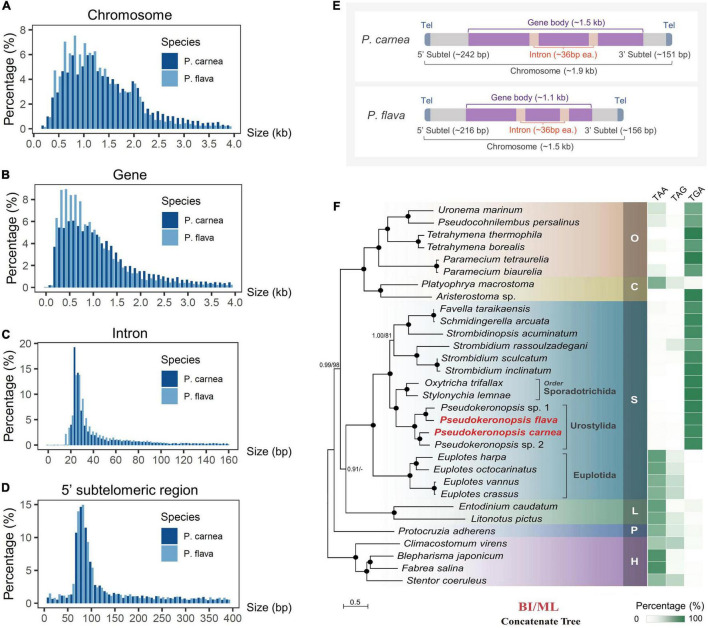
Macronucleus (MAC) genomic features of two *Pseudokeronopsis* ciliates and phylogenomic analysis. **(A–D)** Size distribution of chromosomes, gene bodies (start to stop codon, including introns), introns, and 5′ subtelomeric regions of *P. flava* and *P. carnea* MAC genomes. **(E)** A schematic illustrating the canonical structure of nanochromosomes in *P. flava* and *P. carnea* MAC genomes. Tel, telomere; Subtel, subtelomeric region. The average size of each region is shown in the parentheses. **(F)** Phylogenomic tree (left) and stop codon reassignment (right). Phylogenomic tree is estimated from a concatenated dataset of 238 orthologous proteins by Bayesian inference (BI) and maximum likelihood (ML) methods. Black dots denote full support (BI 1.0/ML 100%). Hyphens denote topological disagreement between ML and BI analyses. O, class Oligohymenophorea; C, class Colpodea; L, class Litostomatea; S, class Spirotrichea; P, class Protocruzia; H, class Heterotrichea; M, class Mesodiniea. The scale bar corresponds to 50 substitutions per 100 amino acid positions.

Using high-throughput sequencing data, we assembled the MAC genomes of two *Pseudokeronopsis* species (Table 1). The genome assemblies of *P. carnea* and *P. flava* are 76.8 Mb and 52.5 Mb in size, respectively, in line with other known ciliate genomes (Figure 1). Most of the contigs bear telomeric repeats (C_4_A_4_) on at least one end (*P. carnea*, 81.1%; *P. flava*, 82.8%). The average size of contigs capped with telomeric repeats on both ends is 1.7 kb (*P. carnea*) and 1.1 kb (*P. flava*), and the vast majority (*P. carnea*, 97%; *P. flava*, 96%) appear to be gene-sized chromosomes (Figures 2A,B), consistent with the nanochromosome architecture found in other spirotrich genomes. Predicted gene numbers in *P. carnea* and *P. flava* are 12,734 and 9,520, respectively. 84% of *P. carnea* and 78% of *P. flava* genes are annotated using SWISS-PROT or NR databases. Introns in these species are tiny (only 36 nucleotides on average for both *P. carnea* and *P. flava*), a feature also observed in several other ciliate genomes (Figure 2C). The subtelomeric regions between the transcription start site (TSS) or transcription end site (TES) and the adjacent 5′ telomere repeat are also short (Figure 2D), similar to previous observations in the nanochromosomes of *Oxytricha*, *Euplotes* and *Strombidium* (Kim et al., 2013; Swart et al., 2013; Chen et al., 2019). Overall, the combined genomic features of *Pseudokeronopsis* represent an extremely compact eukaryotic genome architecture, with single genes containing minimal introns, nested between short subtelomeric regions (Figures 2D,E).

**TABLE 1:** MAC genome assembly information for two *Pseudokeronopsis* species.

	*P. carnea*	*P. flava*
Genome size (Mb)	76.8	52.5
% GC	41.7%	40.4%
Contig	37,909	37,545
% contig with telomere*	81.1%	82.8%
% scaffold (two telomeres)**	38.6%	32.7%
N50 (scaffold)	2,120	1,712
Gene	12,734	9,520
Exon	31,869	21,757

*Percentage of contigs capped with telomere repeats on at least one of two ends.

**Percentage of scaffolds capped with telomere repeats on both ends.

To perform the phylogenomic analysis, we collected public genomic/transcriptomic datasets available for 31 ciliates (Supplementary Table 2), and identified 238 orthologs among *P. carnea, P. flava*, and these species. The system assignment of species we describe here based on maximum likelihood (ML) and Bayesian inference (BI) methods generally agrees with previous studies (Gentekaki et al., 2017; Chen et al., 2018). Phylogenomic analysis of the *P. carnea* and *P. flava* sequenced in the current study shows full support for their cluster with two previously reported *Pseudokeronopsis* species (Figure 2F). The analysis also supports a larger cluster that includes the spirotrichs *Oxytricha trifallax* and *Stylonychia lemnae*. Standard stop codons in ciliates are frequently reassigned to code for amino acids (Swart et al., 2016). Similar to these two species, the stop codons “TAA” and “TAG” are consistently reassigned in all *Pseudokeronopsis* species, leaving “TGA” as the only stop codon (Figure 2F and Supplementary Table 2), reflecting the close evolutionary relationship between these groups. Interestingly, compared to previous phylogenomic analysis result in which the litostomatean *Entodinium* is not included (Chen et al., 2018), we find that the clustering of class Litostomatea and class Spirotrichea is poorly supported in the current BI tree, and not supported at all by the ML tree (Litostomatea clusters with Colpodea first after including *Entodinium*). This opens the possibility that the assignment of litostomateans could require further review in the future by expanding sampling.

### Conserved AT-Rich Sequences Identified as Potential *Cis*-Regulatory Elements

**FIGURE 3:**
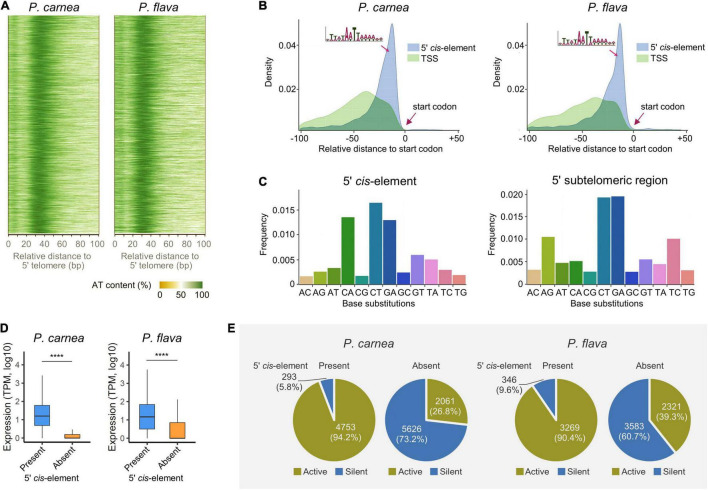
A conserved potential *cis*-regulatory element (CRE) in 5′ subtelomeric region on the nanochromosome. **(A)** Heatmap showing the AT content in the sequences of 5′ subtelomeric regions of *Pseudokeronopsis carnea* (left) and *P. flava* (right). **(B)** Density plot showing the intermediate position of the CRE in an AT-rich motif between transcription start site (TSS) and translation start codon. **(C)** Frequency of base substitutions in 5′ CRE and entire subtelomeric region in *P. flava*. “AC” indicates nucleotide substitutions where adenine is replaced by cytosine, for example. **(D)** The presence of the putative 5′ CRE is associated with the transcription level of its adjacent genes. TPM, transcripts per million. ****p < 0.0001. **(E)** Pie plots showing the percentage of active and silent genes correlated with the presence or absence of the putative 5′ CRE on nanochromosomes.

Although *Pseudokeronopsis* nanochromosomes have limited space, we found an AT-rich region exists in the 5′ subtelomeric regions of most complete nanochromosomes that carry single genes (92.7 and 98.5% for *P. carnea* and *P. flava*, respectively) (Figure 3A). Further analysis 5′ subtelomeric regions reveals a 15 nt putative CRE in the sequence motif W_*n*_AWTW_*n*_ which is positioned between the transcription start site (TSS) and translation start codon in both species (Figure 3B). Similar CREs were identified in the 5′ subtelomeric regions on the nanochromosomes of *Strombidium* (Li et al., 2021) and *Nyctotherus* (McGrath et al., 2007). We identified an additional AT-rich element in the *Pseudokeronopsis* species, this time in the 3′ subtelomeric regions, with the sequence motif “TTNATTTCNTTAA.” This sequence is found between the translation stop codon and the transcription end site (TES) (Supplementary Figure 1A), and is distinct from the 5′ subtelomeric sequence at the other end.

To demonstrate that these potential CREs are conserved evolutionarily, we investigated the base substitution rate of nucleotides in the 5′ subtelomeric regions. The base substitution rate in the putative CRE is 50% lower than that in the entire subtelomeric region (48.2 and 55.2% for *P. carnea* and *P. flava*, respectively), indicating that nucleotides in the CRE are under stronger selection. The substitution pattern also shows a distinct difference between the 5′ CRE and surrounding nucleotides (Figure 3C and Supplementary Figure 1B). Compared with the entire subtelomeric region, the substitution rate of A-to-G and T-to-C is greatly reduced in the CRE, but dramatically increased in C-to-A substitutions, indicating that G/C nucleotides in the CRE are consistently being replaced by A/T nucleotides.

Although not positioned upstream of the TSS, as is seen for TATA-box-like elements in other eukaryotic organisms like yeast (Lin et al., 2010), these sequences may still act as non-canonical regulatory CREs that bind transcriptional trans-activating factors. To determine whether this motif is related to transcription initiation, we compared the expression of genes on nanochromosomes that either possess or lack this CRE in their 5′ subtelomeric regions. We observed that genes with this CRE have significantly higher transcription levels in both species (Figure 3D). The majority of genes without the 5′ CRE are silent and the putative CRE is more associated with active genes, which could be the source of this transcription activity difference (Figure 3E). On the contrary, most of the genes with the adjacent CRE are actively transcribed (94.2 and 90.4% for *P. carnea* and *P. flava*, respectively). These observations suggest that transcription initiation on Pseudokeronopsis nanochromosomes depends on this CRE near the transcription start site, though the nature of this CRE is not clear. The sequence may act as a promoter by binding directly to a transcription factor, or it may contribute a necessary structural feature to the DNA in this region. Future studies should further test whether chromatin accessibility is greater at this location, and if active chromatin marks are enriched (Sheng et al., 2021). A similar association between the 3′ CRE and gene transcription was also identified (Supplementary Figures 1C,D). Together with the conserved base substitution patterns, our results reveal a strong evolutionary selection pressure upon the AT-rich CRE, and suggest it plays a functionally important role in transcription regulation. Considering the compact nanochromosome architecture, the inclusion of these sequences in the primary transcript UTRs, and reassignment of stop codons in these species, it is also possible that these CREs assist in translation initiation/termination at the 5′ and 3′ ends, respectively.

### The Evolution History of Spirotrich Nanochromosomes

**FIGURE 4:**
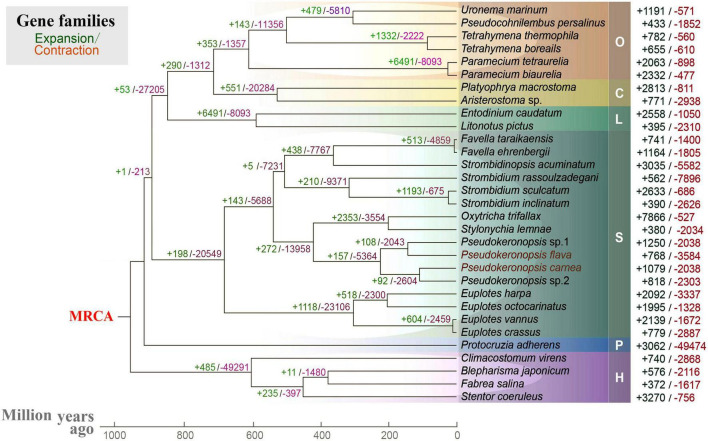
Gene family evolution analysis of 31 ciliates based on 238 orthogroups. Green and red numbers represent the expanded or contracted gene families in each branch, respectively. MRCA, most recent common ancestor. O, class Oligohymenophorea; C, class Colpodea; L, class Litostomatea; S, class Spirotrichea; P, class Protocruzia; H, class Heterotrichea; M, class Mesodiniea.

To help understand the origins of spirotrich nanochromosomes, we investigated the expansion and contraction of gene families in 31 ciliates based on 238 orthogroups. Nanochromosome architecture has been reported in species of several disparate ciliate clades (classes Spirotrichea, Armophorea, and Litostomatea), suggesting multiple origins of extensive fragmentation within ciliates (Riley and Katz, 2001; McGrath et al., 2007; Huang and Katz, 2014; Špaková et al., 2014; Park et al., 2021). The spirotrich clade, which features gene-sized chromosomes, originated approximately 900 million years ago, accompanied by a dramatic expansion/contraction of several gene families (Figure 4). Within spirotrichs, *Pseudokeronopsis* species originated 220 million years ago, which in turn separated from the clade containing *Oxytricha* and *Stylonychia* 430 million years ago. As gene family expansion reflects phenotypic diversity and genetic adaptions during evolution (Harris and Hofmann, 2015; Yan et al., 2019), we identified 1079 and 768 expanded gene families (*p* < 0.05) in *P. carnea* and *P. flava*, respectively. These expanded gene families are enriched in a variety of pathways (Supplementary Figure 2). Compared with three representative species that do not carry nanochromosomes (Supplementary Figure 3), the expanded gene families in both *Pseudokeronopsis* species contribute to transcription factor binding and sequence-specific DNA binding. Although given the incomplete nature of the current ciliate genomic datasets as a limitation, our analyses provide a baseline about the transcription regulation pathways rewiring in species with nanochromosomal organization for the future studies.

## Concluding Remarks

In summary, we report the first macronuclear genome assemblies of two *Pseudokeronopsis* ciliates, which consist of compact, gene-sized nanochromosomes.

Similar to other spirotrichs, *Pseudokeronopsis* nanochromosomes have tiny introns and small subtelomeric regions. We identified AT-rich sequences conserved within the 5′ and 3′ subtelomeric regions in both species and observed that these potential CREs are associated with active gene expression, suggesting a role in transcription regulation. Both *P. carnea* and *P. flava* have expanded their complement of genes related to nucleotide binding and gene expression regulation since the origin of gene-sized chromosomes in spirotrichs approximately 900 million years ago. Together, these findings suggest that ciliates may have developed a unique mechanism to regulate transcription from gene-sized chromosomes during evolution.

## Data Availability Statement

The datasets presented in this study can be found in online repositories. The names of the repository/repositories and accession number(s) can be found below: https://www.ncbi.nlm.nih.gov/, PRJNA507672, PRJNA534036, https://pse.ciliate.org, *Pseudokeronopsis* DB.

## Author Contributions

WZ, CL, XC, and LL conceived the study. WZ provided the biological materials. WZ, WS, and XC designed the experiments. WZ and HD performed the experiments. WZ, CL, HD, and XC performed computational and experimental analysis for all figures and tables. WZ, CL, SAA, WS, and XC interpreted the data. NAS and AB constructed the genome database website. WZ, CL, XC, and LL wrote the manuscript with contribution from all authors. All authors read and approved the final manuscript.

## Author Disclaimer

The content is solely the responsibility of the authors and does not necessarily represent the official views of the National Institutes of Health.

## Conflict of Interest

The authors declare that the research was conducted in the absence of any commercial or financial relationships that could be construed as a potential conflict of interest.

## Publisher’s Note

All claims expressed in this article are solely those of the authors and do not necessarily represent those of their affiliated organizations, or those of the publisher, the editors and the reviewers. Any product that may be evaluated in this article, or claim that may be made by its manufacturer, is not guaranteed or endorsed by the publisher.

## Supplementary Material

Supplementary Figure 1 A potential *cis*-regulatory element (CRE) in 3′ subtelomeric regions of two *Pseudokeronopsis* ciliate genomes. **(A)** Density plot showing the intermediate position of the CRE in a motif “TTNATTTCNTTAA” between the transcription end site (TES) and translation stop codon in 3′ subtelomeric regions of *P. carnea* (left) and *P. flava* (right) genomes. **(B)** Frequency of base substitutions in 5′ CRE and entire subtelomeric region in *P. carnea*. “AC” indicates substitutions where adenine is replaced by cytosine, for example. **(C)** The presence of a putative 3′ CRE is associated with the transcription level of its adjacent genes. TPM, transcripts per million. ^****^*p* < 0.0001. **(D)** Pie plots showing the percentage of active and silent genes correlated with the presence or absence of the putative 3′ CRE on the nanochromosome.

Supplementary Figure 2 Pathway annotation by Gene Ontology (GO) of expanded gene families in spirotrichs: **(A)** *Pseudokeronopsis carnea*, **(B)** *P. flava*, **(C)** *Oxytricha trifallax*, **(D)** *Stylonychia lemnae*, **(E)** *Strombidium sculcatum*, and **(F)** *Euplotes vannus*. Q values (FDR) are indicated by color scale and number of expanded genes in each pathway is indicated by the dot size. Rich factor (as indicated in the *Y*-axis) is the ratio of the number of expanded genes in a pathway to the number of all annotated genes in this pathway.

Supplementary Figure 3 Pathway annotation by Gene Ontology (GO) of expanded gene families in *Tetrahymena thermophila* **(A)**, *Paramecium tetraurelia* **(B),** and *Stentor coeruleus* **(C)**. Q values (FDR) are indicated by color scale and number of expanded genes in each pathway is indicated by the dot size. Rich factor (as indicated in the *Y*-axis) is the ratio of the number of expanded genes in a pathway to the number of all annotated genes in this pathway.
