# Supplementary figures and images for: Comparative Genome Analysis Reveals Cis-Regulatory Elements on Gene-Sized Chromosomes of Ciliated Protists
Source: Front Microbiol. 2022 Feb 21;13:775646. doi: 10.3389/fmicb.2022.775646 (PMC8899921; doi:10.3389/fmicb.2022.775646)

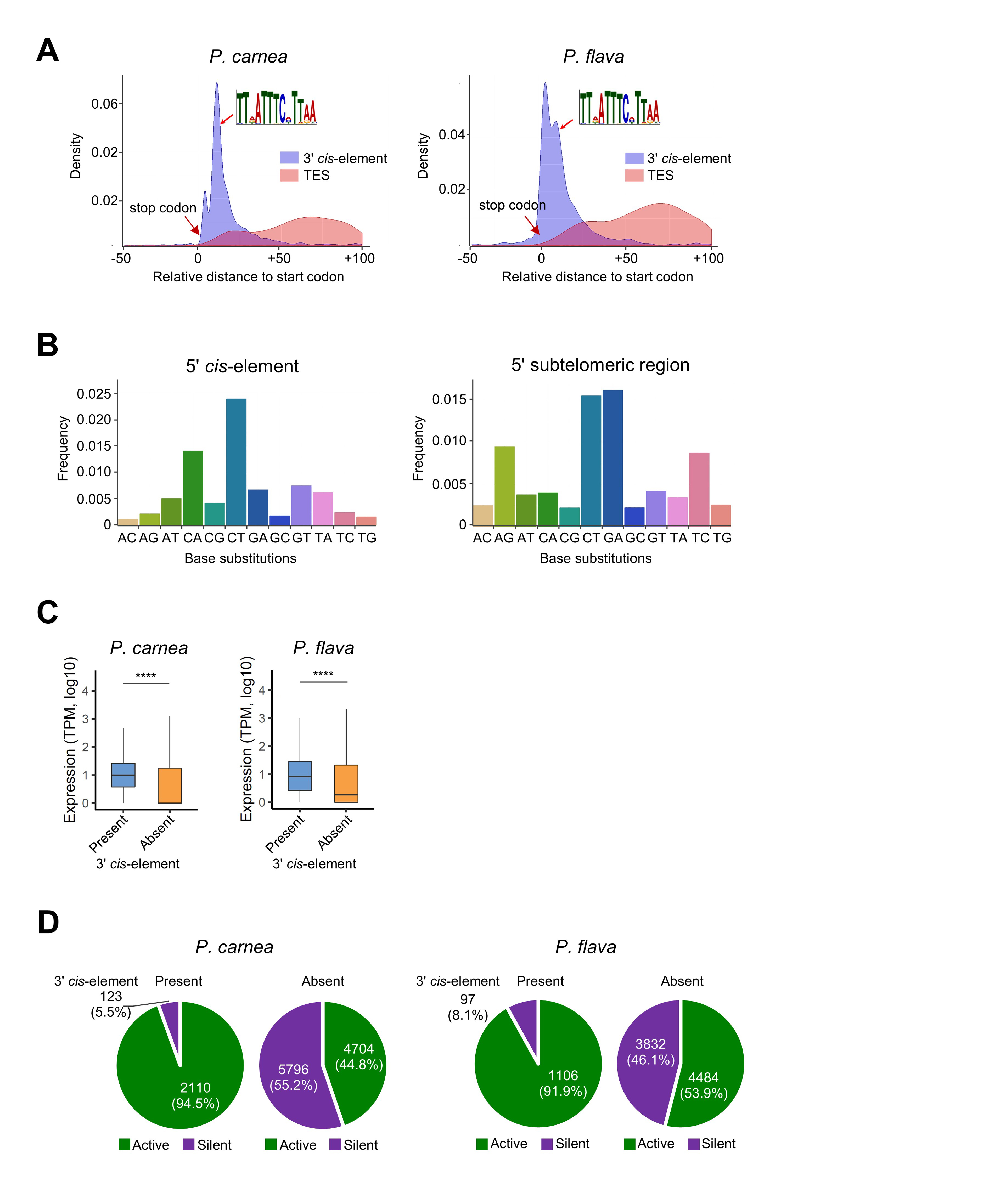

Supplement: Supplementary Figure 1 — A potential cis-regulatory element (CRE) in 3′ subtelomeric regions of two Pseudokeronopsis ciliate genomes. (A) Density plot showing the intermediate position of the CRE in a motif “TTNATTTCNTTAA” between the transcription end site (TES) and translation stop codon in 3′ subtelomeric regions of P. carnea (left) and P. flava (right) genomes. (B) Frequency of base substitutions in 5′ CRE and entire subtelomeric region in P. carnea. “AC” indicates substitutions where adenine is replaced by cytosine, for example. (C) The presence of a putative 3′ CRE is associated with the transcription level of its adjacent genes. TPM, transcripts per million. ****p < 0.0001. (D) Pie plots showing the percentage of active and silent genes correlated with the presence or absence of the putative 3′ CRE on the nanochromosome. [file Image_1.JPEG]

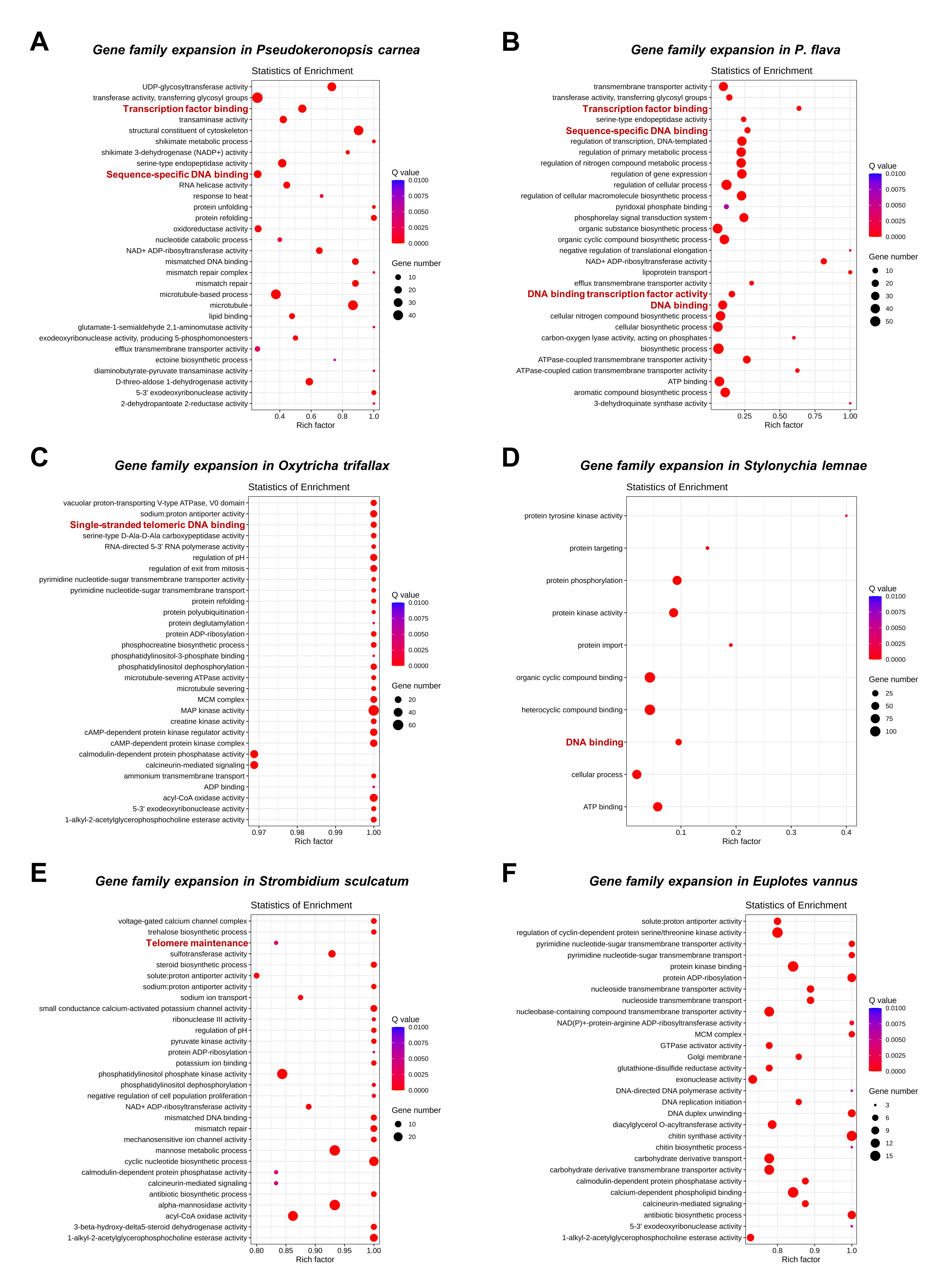

Supplement: Supplementary Figure 2 — Pathway annotation by Gene Ontology (GO) of expanded gene families in spirotrichs: (A) Pseudokeronopsis carnea, (B) P. flava, (C) Oxytricha trifallax, (D) Stylonychia lemnae, (E) Strombidium sculcatum, and (F) Euplotes vannus. Q values (FDR) are indicated by color scale and number of expanded genes in each pathway is indicated by the dot size. Rich factor (as indicated in the Y-axis) is the ratio of the number of expanded genes in a pathway to the number of all annotated genes in this pathway. [file Image_2.JPEG]

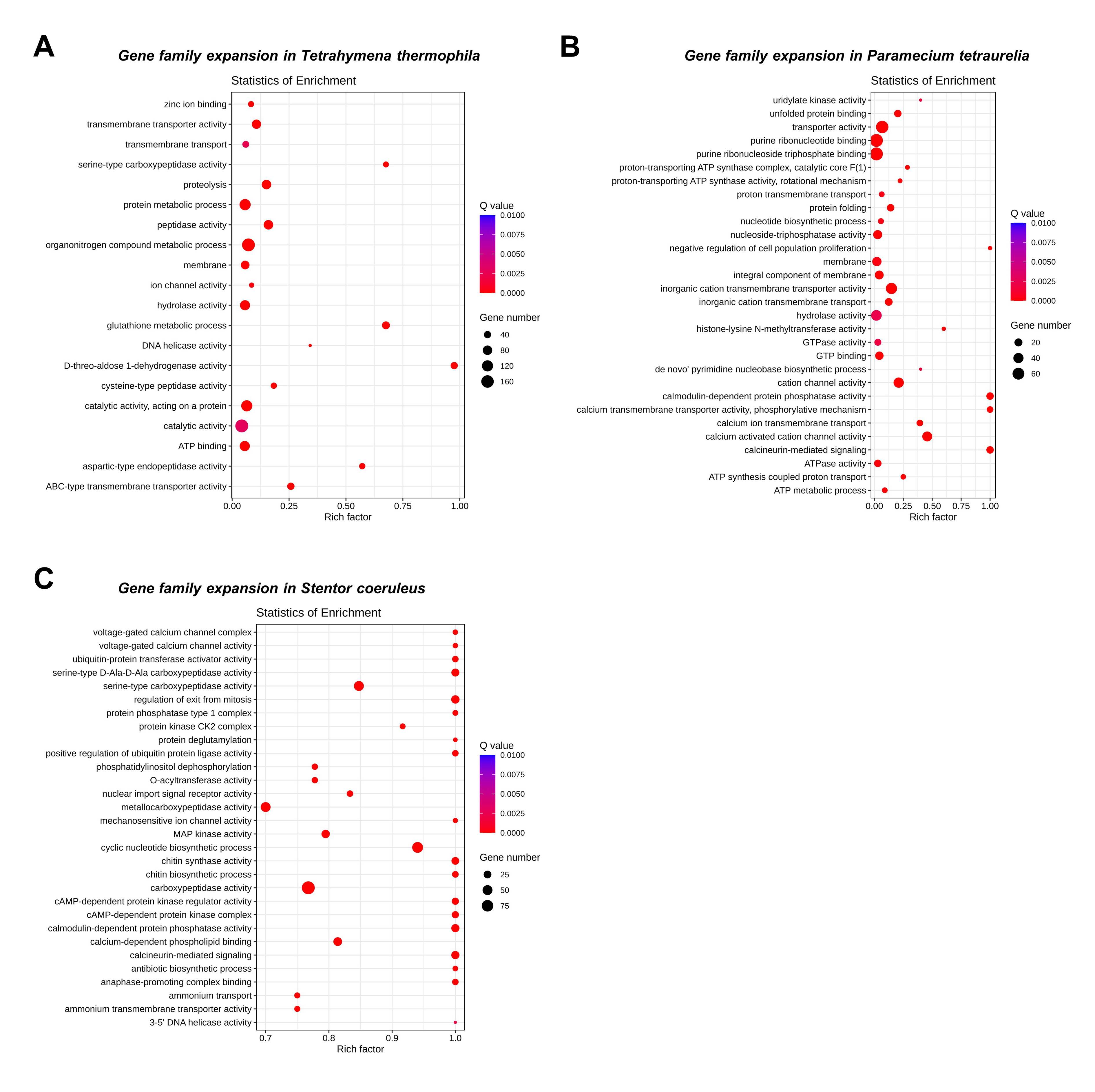

Supplement: Supplementary Figure 3 — Pathway annotation by Gene Ontology (GO) of expanded gene families in Tetrahymena thermophila (A), Paramecium tetraurelia (B), and Stentor coeruleus (C). Q values (FDR) are indicated by color scale and number of expanded genes in each pathway is indicated by the dot size. Rich factor (as indicated in the Y-axis) is the ratio of the number of expanded genes in a pathway to the number of all annotated genes in this pathway. [file Image_3.JPEG]
